# Mind the app: more time spent on headspace leads to beneficial day-to-day changes in mindfulness, depression, anxiety and stress in college students

**DOI:** 10.1080/28324765.2024.2400878

**Published:** 2024-09-09

**Authors:** Mackenzie E. Pierce, Grazia Mirabito, Paul Verhaeghen

**Affiliations:** Psychology, Georgia Institute of Technology, Atlanta, USA

**Keywords:** Mobile apps, mindfulness, anxiety, mechanism

## Abstract

Fifty-seven college students downloaded the Headspace app and gave daily reports of app use, state mindfulness, state depression, state anxiety and state stress over a two-week period. App use was high (86 min). Day-to-day ratings of anxiety and stress decreased, while ratings of state depression and state mindfulness remained stable. Multilevel mediational models showed that spending time on the Headspace app increased an individual’s mindfulness in the moment, which then decreased ratings of stress, anxiety and depression. There was a lagged effect for state anxiety, such that time-on-app on a given day increased mindfulness on the same day, which decreased feelings of anxiety on the next day. The results strongly suggest a sequence where app usage leads to changes in state mindfulness, which are then associated with beneficial changes in mental health. Thus, state mindfulness is a (or perhaps the) key ingredient of the beneficial effects of this mindfulness/meditation app, opening venues for intervention and a potential for cumulative effects.

It is well known that mindfulness interventions have become an increasingly popular and effective method to ease stress, distress, anxiety and depression and to increase well-being, both in clinical and non-clinical populations (for meta-analyses, see Goyal et al., 2014; Gu et al., 2015; Khoury et al., 2013). In non-clinical populations, mindfulness interventions have been shown to enhance attention, to boost positive affect and overall well-being, and to alleviate self-perceived stress, anxiety, depressed mood, negative affect and rumination (for a meta-analysis, see Eberth & Sedlmeier, 2012).

One population that is both in need of and responsive to mindfulness interventions is college students. The need is dire: The 2021–2022 Data Report from the Healthy Minds Study, which surveyed more than 100,000 college students in the US, found that 23% of students screened positive for major depression, 37% screened positive for anxiety disorder and 11% experienced trauma and/or stress-related disorders. In a relatively recent survey conducted at the institution where we conducted our research, 91% of students stated being “very stressed” (Singleton & Hale, 2017). Mindfulness interventions appear to be one effective form of treatment in this population, with effect sizes of Hedges’ *g* = 0.47 for depression and Hedges’ *g* = 0.58 for anxiety (effect sizes from a meta-analysis by Ma et al., 2022; Hedges’ *g* is the mean standardized difference between intervention and control treatment adjusted for sample size). Mindfulness interventions aimed at college students also appear to be effective in delivery modes other than the standard in-person group intervention. For instance, online interventions lead to significant (but possibly reduced) effect sizes for depression, Hedges’ *g* = 0.27, and anxiety, Hedges’ *g* = 0.47 for anxiety and are also effective at curbing stress, Hedges’ *g* = 0.58 (effect sizes from a meta-analysis by Gong et al., 2023).

Meditation and mindfulness apps are another alternative mode of delivery for mindfulness interventions. Such apps have the advantage that they can be distributed widely and easily at a relatively low cost, with a very low threshold for participation. In the general public, such apps have been found to beneficially and significantly impact levels of anxiety (Hedges’ *g* = 0.28), depression (Hedges’ *g* = 0.33), perceived stress (Hedges’ *g* = 0.46) and overall well-being (Hedges’ *g* = 0.29) (effect sizes from a meta-analysis by Gál et al., 2021). A systematic review comparing the efficacy of Headspace and Calm (the two most popular mindfulness apps in 2022) found that 40% of studies using Headspace reported an improvement in well-being and anxiety, and 75% of Headspace studies improved depressive symptoms (O’Daffer et al., 2022). A perhaps obvious prerequisite for this is that participants actually use the app (e.g., Flett et al., 2019; Parsons et al., 2017).

A crucial question for any intervention concerns the mechanism through which it operates. The proposed key mechanism for in-person and online mindfulness interventions is mindfulness (for a review of models asserting this, see Vago & Silbersweig, 2012). Therefore, mindfulness curricula often and extensively emphasize the practice and/or conceptual background of mindful awareness. Testing such mechanisms is usually done through mediation analysis, where it is tested whether the strength of the influence of a predictor (such as using a mindfulness app) on the outcome (such as mental health) is significantly reduced when the influence of the mediator (e.g., mindfulness) is statistically controlled for (e.g., Baron & Kenny, 1986). Research has indeed indicated that mindfulness is a crucial (and likely causal) ingredient for mindfulness-based interventions. In their 2015 meta-analysis, Gu et al. found that changes in trait mindfulness after a mindfulness intervention explained on average 33% of the variance in stress, anxiety, or depression (12 studies).

The estimates from Gu et al. (2015), however, are based on two-point (i.e., pre-post intervention) measurements, that is, without intermediate assessments of mechanisms or outcomes. The hypothesis of mechanistic causality would, of course, be greatly strengthened by establishing the existence of a temporal sequence between predictors, mediators and outcomes through more frequent assessment. Parts of the sequence have been established previously—in intervention studies examining day-to-day variations in mindfulness through ecological momentary assessment (typically daily surveys at unexpected times, administered through the participants’ smartphones), mindfulness has been found to decrease momentary negative affect (Brown & Ryan, 2003), to increase momentary positive affect (Geschwind et al., 2011; Shoham et al., 2017), or both (Gotink et al., 2016; Quaglia et al., 2015), as well to decrease emotional lability (Quaglia et al., 2015) and to decrease symptoms of depression and increase well-being (Mirabito & Verhaeghen, under review). As far as we know, no studies have explicitly taken a full mediation approach, that is, one where app use leads to state changes in mindfulness, which, in turn, lead to changes in mental health.

Note that in studies using state measures, that is, assessments of mindfulness and proposed treatment outcomes as they are experienced in the moment, the locus of causality is firmly placed at the intraindividual level, where it arguably belongs, at least from a salutary or therapeutic point of view. Traditional pre-post designs use trait measures (i.e., measures of more or less stable interindividual differences) and hence place this locus at the interindividual level (i.e., “Will people who use the app longer or more often become more mindful and thereby feel happier?”). People who choose to go through a mindfulness intervention, we presume, are primarily interested in effects at the intraindividual level we investigate here (i.e., “After I use the app, will I feel more mindful and happier?”). As far as we know, no studies have utilized a frequent-assessment or daily-diary design focusing on meditation apps.

A major drawback of mindfulness and meditation apps is that app usage typically declines over time (e.g., Flett et al., 2019; Levin et al., 2022; Parsons et al., 2017). Relatively little is known about the reasons for this. One study, not using an app but an in-person mindfulness intervention, found that baseline conscientiousness and openness predicted intervention adherence (Canby et al., 2020). Adherence to practice is important because it directly impacts the effectiveness of mental health outcomes (Parsons et al., 2017).

In the present study, our primary aim was to examine (as far as we know, for the first time) the mechanisms of change after app use—whether day-to-day use of a mindfulness app leads to day-to-day increases in state mindfulness and whether these changes, in turn, are linked to day-to-day changes in mental health (here: depression, anxiety and stress) over the course of two weeks. Our hypothesis is mediational: We hypothesize that time spent on the app will lead to higher levels of state mindfulness, which in turn will lead to lower levels of depression, anxiety and stress, thus aiming to identify mindfulness as the mechanism through which meditation translates itself into positive mental health outcomes. The app we chose was Headspace, first, because it is the app that has been studied most frequently (Gál et al., 2021; O’Daffer et al., 2022). Second, it is generally well liked: In a review of mindfulness apps, Headspace was the mindfulness app that scored highest on the Mobile Application Rating Scale (Mani et al., 2015), a scale focusing on engagement, functionality, aesthetics, informational and consumer satisfaction of the app. Third, this app was available to all students on our campus for free, through a program from Student Life, which means that participants would be able to continue using the app after the study if they so desired. Note that because this study concerns the coupling of within-person changes in people who use the app, participants serve as their own controls and no control group is necessary (or even feasible) for these mediational analyses. Our secondary aim was to examine predictors of adherence. To that aim, we included personality measures (the Big Five) as well as baseline mindfulness (operationalized as the mindfulness manifold of reflective awareness, controlled sense-of-self in the moment, self-preoccupation, self-compassion and self-transcendence; Verhaeghen, 2019), baseline mental health and prior experience with mediation and mindfulness or meditation apps as our predictors of drop-off. We consider this secondary aim exploratory. Because we were collecting baseline measures of trait mindfulness and mental health for the adherence analysis, we decided to also include these measures (as well as an instrument to rate participants’ satisfaction with the app itself) in a posttest. This allowed us, as a tertiary aim, to explore whether scores on these trait measures changed and whether changes in state and trait measures were correlated. Given that effects of mindfulness apps are typically already of small magnitude for programs with a median duration of four weeks (*g* = 0.36 on average for anxiety, depression and stress in a meta-analysis by Gál et al., 2021), we did not necessarily expect significant results on trait measures in our two-week trial.

## Methods

### Participants

The final sample consisted of 57 students. They participated in return for course credit (53% male, 46% female; average age 19.48; 58% Asian or Asian-American, 33% White; 11% African-American; 1% Hispanic). The study was approved by the Georgia Institute of Technology Institutional Review Board as protocol H22497 with a waiver of documentation of consent (i.e., because the study was conducted online, participants indicated consent with a button press instead of an actual signature). Participants were recruited from introductory psychology classes for “a study on how people can learn mindfulness through a phone app”. To be included, participants needed to be fluent in English and 18 years of age or older. Eight more students signed up but did not complete the post-test and were thus not included in the analyses. There were no significant differences between the group of participants who participated in the posttest and those who did not on any of the demographics or other measures gathered at pretest, largest *t*(63) = 1.26, *p* = 0.21. Forty-three percent of the participants indicated they had used a meditation or mindfulness app before; of those, 71% indicated having used an app “a few times (less than ten times)”; 21% indicated having used an app “between ten and fifty times”; and 3% indicated having used an app “more than fifty times”. Apps used were Calm (54% of those who had used an app before), Headspace (43% of those who had used an app before), Insight Timer (4% of those who had used an app before) and “other” (21%; percentages add up to a number larger than 100% because participants were allowed to indicate more than one app).

### Procedure

The entirety of the study was conducted online and/or via smartphone. After signing up for the study, the participants received their individualized participant number and a link to the pre-test survey. After completing the first survey, the participants were instructed on how to download Headspace and the Expiwell application, which was used for administering and recording the daily surveys, on their smartphones. They were asked to use the Headspace app at their own discretion over the course of the next two weeks. Once daily, at 6 pm, they were prompted by the Expiwell app to fill out the daily surveys about app use, state mindfulness, depression, anxiety and stress. The daily surveys were available to be completed from 6 pm to midnight. At the end of the two-week period, the participants took the post-test survey. The pre- and post-surveys took approximately 45–60 min, and the daily surveys took 1–2 min to complete. Credit awarded for the study was only tied to completion of the pretest and post-test surveys, not to app use or the number of daily surveys responded to, so that participants could feel free to use the app as much or as little as they wanted, or not at all.

### Measures

Our primary interest was in daily measures of app use, state mindfulness and state mental health. Additionally, we included subjective measures of app quality, and pre-post trait measures of trait mindfulness, mental health and personality.

### App use and daily state measures

Every day (see Procedure) participants were asked whether they had used the *app* since the last survey, and, if so, they indicated how long, how often, what app functions they used (meditate, move, sleep, music and/or other) and duration of engagement. These surveys were administered through a smartphone app, ExpiWell, on the participants’ personal smartphones. They also indicated on a slider scale (score 1–100, with 1-point increments) how *mindful*, *depressed*, *anxious* and *stressed* they felt. Traditionally, single-item measures have suffered a bad reputation, both from a reliability and a validity standpoint of (for a review, see Allen et al., 2022). From a classical psychometric standpoint, aggregating items is assumed to help canceling out random measurement error contained within each item (Shrout & Lane, 2012). Single-item measures, however, have obvious pragmatic advantages, especially in a daily-assessment context (Allen et al., 2022). For EMA, some have recommended assessing complex constructs with at least three items, while discrete phenomena or behavior may be assessed with a single item (Trull et al., 2020). Empirically speaking, single-item measures have been found to reliably and validly measure the constructs assessed here, namely mindfulness (Meier et al., 2023), anxiety (Davey et al., 2007; Turon et al., 2019; Young et al., 2015), depression (Ayalon et al., 2010; Huang et al. 2020; Song et al., 2023; Turon et al., 2019; Young et al., 2015) and stress (Arapovic-Johansson et al., 2017; Elo et al., 2003; Karvounides et al., 2016; Littman et al., 2006). As a validity check on our state measures, we investigated their relationship with the trait variables in a confirmatory factor analysis reported below.

### App rating

The Mobile Application Rating Scale (MARS; Terhorst et al., 2020) measures subjective app quality with subscales for Engagement (how fun and interesting participants judge the app to be), Functionality (how well the app functions, how easy it is to navigate and so on), Aesthetics (overall appeal) and App Subjective Quality (whether participants would recommend the app to others, be willing to pay for it, and whether they think they will use it in the future). This rating was, for obvious reasons, only administered during the post-survey.

### Pre- and post-survey trait measures

The short version of the Depression Anxiety Stress Scale (DASS), the DASS-21 (Henry & Crawford, 2005), assesses *depression*, *anxiety* and *stress* over the past week.

*Mindfulness* was measured as the five-part mindfulness manifold as derived after a set of exploratory and confirmatory factor analyses as reported in Verhaeghen (2019). *Reflective awareness* was measured as the unit-weighted *z*-score composite of three questionnaires: (a) the Observing subscale of the Five Facets Mindfulness Questionnaire (FFMQ; Baer et al., 2008; 8 items); (b) the Reflectiveness subscale of the Broad Rumination Scale (BRS; Trani et al., in preparation; 4 items); and (c) Search for Insight/Wisdom of the Aspects of Spirituality scale (ASP; Büssing et al., 2007, p. 7 items). *Controlled sense-of-self in the moment* was measured as the unit-weighted *z*-score composite of three questionnaires: (a) the Acting with Awareness subscale from the FFMQ (eight items); (b) the Sense-of-self Scale (SOSS; Flury & Ickes, 2007, p. 12 items); and (c) the Nonjudging of inner experience subscale of the FFMQ (eight items). *Self-preoccupation* is the unit-weighted *z*-score composite of two subscales from the BRS, namely Compulsivity (five items) and Worrying (three items), and the Isolation (two items) and Over-Identified (two items) subscales of the Self-Compassion Scale, Short Form (SCS; Raes et al., 2011). *Self-compassion* was measured as the unit-weighted *z*-scores composite of the Self-Kindness (two items), Common humanity (two items) and Mindfulness (two items) subscales of the SCS, as well as the Decentering subscale of the Experiences Questionnaire (EQ; Fresco et al., 2007, p. 13 items). *Self-transcendence* was measured as the unit-weighted z-score composite of the Joy (six items) and Love (six items) subscales of the Dispositional Positive Emotion Scale (DPES; Shiota et al., 2006), and the Meaningfulness (seven items) subscale from the Resilience Scale (RS; Lundman et al., 2007).

*Personality factors* were assessed using the Mini IPIP (Donnellan et al., 2006), a validated 20-item shortened scale of the International Personality Item Pool, measuring the Big Five personality traits: Openness to Experience, Conscientiousness, Extraversion, Agreeableness and Neuroticism.

We also collected *demographic information* and asked questions about *prior experience* with meditation and with meditation and/or mindfulness apps.

### Data analyses

Changes in the state variables were examined using multilevel restricted maximum likelihood regression analysis through the *lme* function of the R package *nlme* (version 3.1–143; Pinheiro et al., 2022). Specifically, the nesting has daily repeated measures (level 1) nested within a person (level 2). Note that MLM time series analyses are robust against missing data and therefore missing data were excluded (Snijders & Bosker, 2012). Our sample had 24% of the total possible 812 daily data points missing.

We tested for multilevel mediation with the mediation effects at level 1, setting time spent on the Headspace app as the predictor, state mindfulness as the mediator, and state stress, state anxiety and state depression as the outcome variables. Multilevel restricted maximum likelihood moderated mediation analyses were performed using the *lme* function of the R package *nlme* (version 3.1–143; Pinheiro et al., 2020). All variables were centered within-person; the variables were stacked to allow dummy codes to represent whether a variable is an outcome or a predictor. Models were run both with and without autocorrelation. Mediation was determined by Arioan’s test (Aroian, 1976), a two-tailed *z*-test that is a less conservative alternative to the Sobel test. In our analyses, we implemented different time (*t*) lags between the mindfulness variable, mediators and outcome variables. Essentially, we investigated over what time lag app use was able to predict changes in the mediator variable (e.g., time-on-app on day *t* predicting mindfulness on day *t* +1, *t* +2 and so on) and over what lag the mediator was able to predict changes in the outcomes (depression, anxiety and stress).

## Results

### App usage

Participants were prompted once daily to provide information on their usage of the app. Over the course of the study, there were thus 14 prompts. On average, participants replied to 10.53, or 76%, of the prompts (*SD* = 3.48, median = 12). Our participants reported, on average, using the app 0.92 times per prompt answered (*SD* = 0.70, median = 1), for an average duration of 8.56 min (*SD* = 9.92, median = 10). Total time spent on the app over the course of the two weeks was on average 87.02 min (*SD* = 94.00; median = 60).

The most often accessed function of the app was its meditation function (74% of prompts), followed by the sleep function (20% of prompts) and the music function (15%), with the move function used least often (10%); “other” functions were accessed even less often (7%). Note that these percentages add to more than 100% because participants could indicate more than one function for each prompt, given that they made have accessed the app more than once. If we calculate percentages over the total number of mentions of any function, the meditation function was accessed in 59% of all instances, sleep in 16%, music in 12%, the move function in 8% and “other” in 6%.

In 42% of the prompts, the app was accessed in the evening; morning access was at 33%; afternoon access at 31%; for 22% of prompts the app was accessed right before bed. Note that percentages add up to more than 100% because participants might have accessed the app more than once for each prompt. Calculated over the total number of mentions, these percentages are 33%, 26%, 24% and 17%, respectively.

### State measures: relationship to trait constructs

To assess whether the state and trait constructs were related, we performed a confirmatory factor analysis (CFA using ML estimation) using the pre- and post-scores on the FFMQ subscales as the trait measures of the mindfulness construct as traditionally defined, and the pre and posttest relevant subscales of the DASS-21 as trait measures of anxiety, depression and stress. This priori model fit the data adequately but with a large amount of residual variance, chi-square (*df* = 164) = 584.71, GFI = 0.96, SRMSR = 0.19. Modification indices suggested that freely estimating the residual covariances between the three mental-health state variables (possibly due to shared method variance, shared construct variance, or both) would considerably improve model fit. This model fit better than the original, delta chi-square (df = 3) = 99.00, *p* < .001. Fit for this model was chi-square (*df* = 161) = 485.62, GFI = 0.97; SRMSR = 0.172. Factor loadings for this model are presented in Table 1. The factor loadings suggest that the state anxiety, depression and stress measures all share variance with their respective trait measures, underscoring the validity of the state measures. For mindfulness, some non-significant loadings emerged, suggesting that the state measure shares variance with the Acting with Awareness, Nonjudging and Describing aspects of trait mindfulness but not with Observing and Nonreactivity.

**Table 1.** Factor loadings from confirmatory factor analysis on state and trait variables

Factor	Indicator	Estimate
Mindfulness	State mindfulness	4.70*
	Pre FFMQ observing	0.06
	Pre FFMQ describing	0.17*
	Pre FFMQ acting with awareness	0.61***
	Pre FFMQ nonjudging	0.27**
	Pre FFMQ nonreactivity	0.13
	Post FFMQ observing	0.11
	Post FFMQ describing	0.11
	Pre FFMQ acting with awareness	0.61**
	Post FFMQ nonjudging	0.36***
	Post FFMQ nonreactivity	0.14*
Anxiety	Pre DASS-21-21 anxiety	0.52***
	Post DASS-21 anxiety	0.54***
	State anxiety	4.17*
Stress	Pre DASS-21 stress	0.57***
	Post DASS-21 stress	0.53***
	State stress	3.57*
Depression	Pre DASS-21 depression	0.58***
	Post DASS-21 depression	0.52****
	State depression	4.51*

Note. *p < .05, **p < .01, ***p < .001.

### State measures: growth curve analysis

**Figure 1.**
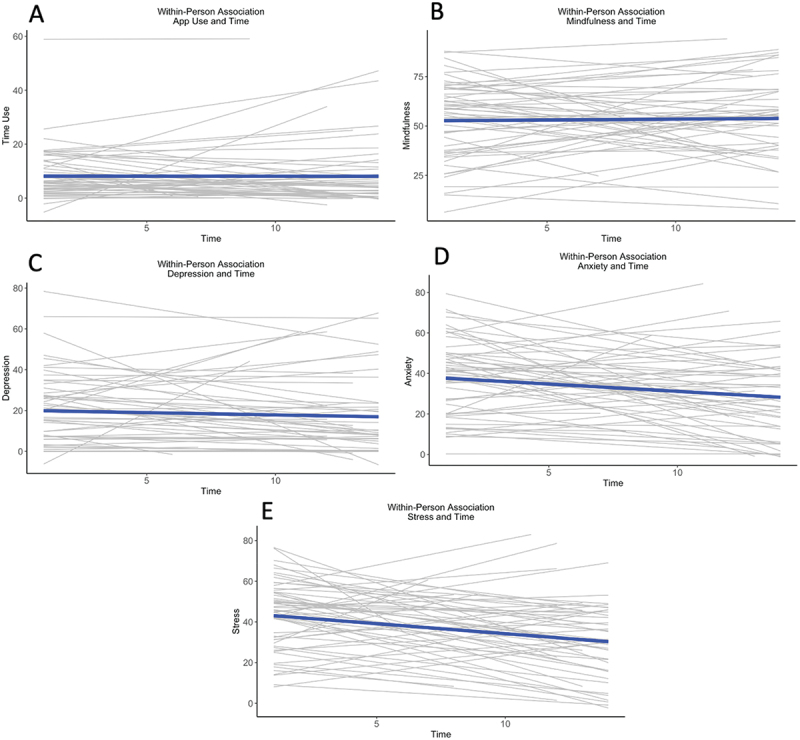
Time course of effects in app use and state variables (fixed effects). The grey lines represent each individual participant’s slope over time, the blue line the average slope. Average slope was significantly different from zero for anxiety (panel D) and stress (panel E).

Results for the MLM growth-curve analysis for the EMA variables are shown in Figure 1. All analyses were first performed specifying autocorrelation. However, for none of the five variables did the model that specified autocorrelation fit better than the model that did not; therefore, we defaulted back to the more parsimonious model of no autocorrelation. To test for non-linear trends, models adding a quadratic term for time were applied to all variables as well. None of these analyses showed evidence for non-linearity (lowest *p*-value for quadratic component = 0.16).

The thick lines in the spaghetti plot in Figure 1 represent the fixed effects, that is, the average results for a typical participant. Change over time was significant for anxiety (slope = −0.71, *p* = 0.00) and stress (slope = −0.92, *p* < .001) (both survived Bonferroni correction), but not time-on-app (slope = 0.01, *p* = 0.94), mindfulness (slope = 0.12, *p* = 0.60) or depression (slope = −0.25, *p* = 0.18).

### State measures: mediational analyses

**Figure 2.**
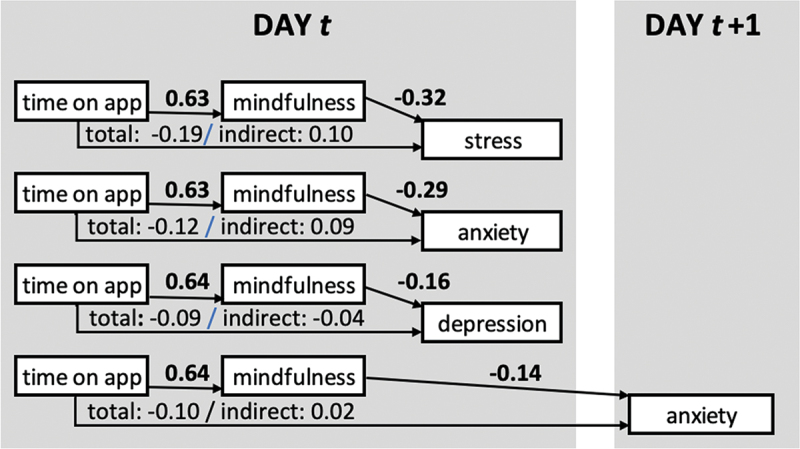
Results of mediational analyses (only models with significant mediation effects are shown). All coefficients are standardized; boldfaced coefficients are significant at *p* < 0.05.

The first set of analyses concerned within-day mediation. We investigated whether self-reported state mindfulness might mediate the relationship between time-on-app and self-reported depression, anxiety and stress within the daily prompts. This was indeed the case, as indicated by Aroian tests; Figure 2 depicts the standardized paths and coefficients. Time spent on the app was associated with higher levels of state mindfulness, which in turn was associated with decreased ratings of state stress, state anxiety and state depression (Aroian tests = 2.80, *p* = 0.00; 2.75, *p* = 0.01; and 2.39, *p* = 0.02; resp.).

In a second set of analyses, we examined lagged relationships. The relationship of time-on-app to mindfulness was not significant for any of the next-day models. Examining models where mindfulness (linked to time-on-app as reported during the same prompt) was associated with outcomes on the next day yielded one significant model, namely the one involving state anxiety (Figure 2) (Aroian test for depression = 0.20, *p* = 0.84; for anxiety: −2.14, *p* = 0.03; for stress: −1.39, *p* = 0.16). No lagged effects extended beyond this one-day delay.

### App ratings

On the MARS survey, participants rated the app on average 3.60 (SD = 0.58) for engagement, 4.20 (*SD* = 0.54) for function, 4.21 (*SD* = 0.49) for aesthetics and 3.04 for subjective quality (SD = 0.73) (the Likert scale alternatives ranged from 1 to 5, with 5 indicating the most positive score). Total time-on-app correlated positively with the two former MARS variables (*r* = 0.44, *p* = 0.00, and *r* = 0.28, *p* = 0.04, resp.), but not aesthetics, *r* = 0.24, *p* = 0.08, or subjective quality (*r* = −0.02, *p* = 0.88).

### Correlates with app usage

Correlations between pre-test measures and prior meditation or app use history with Headspace use in the present study (we examined both total time-on-app and total number of times the app was accessed) were largely non-significant, with the exception of the correlation between total time on app and the baseline DASS-21 Depression score (*r* = 0.25, *p* = 0.04), the correlation between total-time on app and the baseline DASS-21 Anxiety score (*r* = 0.27, *p* = 0.03) and correlation between total-time on app and the baseline Self-Transcendence composite (*r* = −0.36, *p* = 0.00). None of those correlations survived Bonferroni correction.

### Pre-post effects

Only one of our outcome variables showed significant change from pretest to posttest, namely the DASS-21 Depression subscale (mean pre = 1.88; *SD* pre = 0.67; mean post = 1.74; *SD* post = 0.59; t(53) = 2.45; *p* = 0.02; Hedges’ *g* = 0.22), but this effect did not survive Bonferroni correction. The nominal decreases in DASS-21 Anxiety (mean pre = 1.91; *SD* pre = 0.60; mean post = 1.88; *SD* post = 0.67; t(53) = 0.38; *p* = 0.71; Hedges’ *g* = 0.04); and DASS-21 stress (mean pre = 2.06; *SD* pre = 0.63; mean post = 2.02; *SD* post = 0.63; t(53) = 0.79; *p* = 0.44; Hedges’ *g* = 0.07) were very small and not reliable. Note that our sample size of 57 yields a power of 0.96 to detect a medium-sized effect for a paired-sample *t*-test (i.e., Hedges’ *g* = 0.50; alpha = 0.05).

## Discussion

The main impetus for our research was the question whether day-to-day use of a mindfulness app (Headspace) would lead to day-to-day increases in state mindfulness and whether these changes, in turn, would be linked in day-to-day changes in mental health. Additionally, we investigated whether app usage could be predicted from a host of individual-difference variables.

In our within-day analyses, we found that spending time on the Headspace app, as predicted, increased an individual’s mindfulness in-the-moment, which then in turn decreased ratings of stress, anxiety and depression. For state anxiety, this cycle reliably replicated across days such that time-on-app on a given day increased mindfulness on the same day, which decreased feelings of anxiety on the next day.

These findings speak directly to the mechanism. First, the lagged analysis establishes the temporal direction of influence between mindfulness and anxiety: Higher levels of mindfulness beget lower levels of anxiety, with a waning effect over a 24-h period as observed in the decrease of the mindfulness-anxiety coefficient from −0.32 for within-day mediation to −0.14 for across-day mediation. Second, our design also allowed us to establish a temporal direction from app use to mindfulness and the mental health variables. That is, at each prompt, participants were asked about app use in the past (viz., “in the time since the last survey”), whereas the prompts for state mindfulness, anxiety, stress and depression referred to the present, thus creating a natural lag between app use and the state variables. The last survey typically occurred 24 h prior, but, given that participants did not always respond to all prompts, this time frame could also be 48 h or longer. Thus, we can establish that app use at some previous point in time leads to higher mindfulness in the present moment, which leads to lowered anxiety at a later time point. For depression and stress, the model is compatible with mediation through mindfulness, but the temporal across-day lag between mindfulness and these variables necessary to univocally ascertain the temporal sequence was not present.

It is not immediately clear why only anxiety showed a delayed effect. One reason could be that all mindfulness-related effects likely fade out over time (e.g., Carson et al., 2004, found in a mindfulness intervention that the efficacy of mindfulness practice on different measures of relationship quality declined over days). The second-day effect on anxiety (which has the strongest within-day relationship with mindfulness) might have just happened to be strong enough to survive above the threshold for significance. Future research could examine this hypothesis by increasing either statistical power (by including more participants) or temporal precisions (by including more measurement points), or both. One potential rival explanation could be that anxiety might be an easier target for intervention, for instance because it is more volatile than stress or depression. To examine this possibility, we tested for differences between within-person variability (operationalized as within-person variance) for the three outcomes. Anxiety did have reliably larger within-person variance than depression (variance = 367.56 and 175.87, resp., *t*(56) = 6.53, *p* < 0.001), but not stress (variance = 392.26, *t*(56) = −1.19, *p* = 0.24 in the direction against the hypothesis), making this explanation less likely. Another potential reason why a particular variable might be more amenable to change is if it is further removed from the measurement floor. This too was not the case: Average initial scores on the stress scale were higher than those on the anxiety scale, as can be seen in Figure 1.

These results should be placed in the context of changes over time in state variables. Over the course of the study, day-to-day ratings of anxiety and stress decreased, while ratings of state depression and state mindfulness remained stable. The beneficial changes in anxiety and depression cannot be unambiguously ascribed to app use, because our study did not include a control group. We do note, however, that the data shown in Figure 2 suggest (a) that a causal sequence from app use through mindfulness to anxiety and stress is extremely plausible, and (b) that, if we accept point (a), the effects of app use might ultimately be cumulative, such that the day-to-day decline in self-reported stress and anxiety occurs even while app use and mindfulness remain stable, likely through a combination of within-day and delayed effects.

One possible issue with self-report data like ours is that such results may be at least partially explained by the possible presence of response bias. That is, participants signing up for and going through a mindfulness intervention might be inclined to report beneficial intervention effects, just by virtue of the experimenter demands or participant expectations associated with the intervention. We cannot exclude such effects with our present design, but we think these are unlikely, for three reasons. First, the effects on day-to-day measures are relatively subtle (see Figure 1), with only small changes over time, which might be hard for participants to fabricate. Second, if response bias were at work, it would need to be explained why it only operated for some of our variables, and not for others. Notably, we found no effect on mindfulness, which, in a mindfulness intervention, would perhaps be most likely to be subject to demand and/or expectation effects. Third and foremost, our main results concern an analysis of correlations and mediation effects built from day-to-day changes, and those results would seem exceedingly difficult for participants to purposefully or unconsciously generate.

One reason for our study was to examine the precursors of app usage. We were, however, not successful at finding predictors of app use. There are hints that those who stand to benefit more—students who were initially more depressed and more anxious and experienced less self-transcendence—used the app more often, but those associations did not survive Bonferroni correction.

An important point of note is that the trajectories for trait (i.e., pre-post) and state (i.e., day-to-day) variables did not agree: In the pre-post comparison on trait variables, only depression improved; in the day-to-day analysis of state variables, only anxiety and stress improved. A typical assumption in intervention research is that changes in state variables will lead to altered trait variables. An earlier mindfulness intervention study found a similar discrepancy between state and trait effects (Mirabito & Verhaeghen, 2023): There were significant changes in state measures of depression and well-being, but not in their trait version. One possibility Mirabito and Verhaeghen entertained was that the nomothetic span of state and trait variables might differ, that is, state and trait might, in fact, tap into different constructs. In our CFA, we found that state depression, anxiety and stress shared variance with their trait counterparts; state mindfulness appeared to be a narrower construct than traditional definitions of mindfulness, notably missing the observing and non-reactive facets. Additionally and/or relatedly, it is likely that there is extensive interindividual variability in people’s definition and/or phenomenology of what it means to be mindful, depressed, anxious, or stressed. While this is a drawback from a traditional psychometric point of view, we argue that, by their very open-ended nature, state variables such as ours have high ecological validity for each of our participants and might thus possibly be the best target for an individual-centered intervention.

Another possible reason for the state-trait discrepancy could be that trait variables might be subject to memory biases, whereas state variables are not. For instance, when asked to indicate how much a statement from the DASS-21 such as “I tended to over-react to situations” applied over the last week, participants’ judgments might be subject to availability bias (i.e., how quickly they can find a good instance of overreacting within the probed period) and they might thereby overestimate or underestimate the occurrences of such instances. In contrast, state questions do not require aggregation over time and are thus less subject to such biases.

A third possible reason might be that trait assessments are, by design, geared towards stability (e.g., in the appraisal of their reliability) and maximize the measurement of interindividual (not intraindividual) differences (Draheim et al., 2019). These psychometric characteristics make trait measures less amenable to change than state measures are.

Finally, and importantly, a fourth explanation could be that the assumed state-to-trait transition takes longer than the two weeks of app use provided here (or the four weeks in Mirabito & Verhaeghen, 2023). It is unclear from the literature when that transition might happen. The Gál et al. (2021) meta-analysis on the effects of apps found small-to-medium effects with a median intervention duration of 4 weeks, but unfortunately these authors did not investigate dose–effect relationships within their sample of studies.

Our findings allowed us to identify a traceable and sound mechanism behind short-term changes after app usage. This, by implication, suggests that more frequent or longer app use should be encouraged. Our participants did use the app relatively frequently—about ten times over the course of two weeks, for a total time of 86 min. Surprisingly, time-on-app was stable over the course of the study, and so (perhaps concomitantly) was participants’ rating for state mindfulness. In an intervention like this one, one desired outcome would be that app use increases over time. Many app-based interventions find, on the contrary, that app usage declines over time (e.g., Flett et al., 2020; Parsons et al., 2017). Thus, the stability that was obtained here can be seen as a success. App use in the present study was much higher, for instance, than in a recent large-scale study on first-year students, where participants accessed the app on average 7.9 times over the course of three months (Flett et al., 2020); total time spent on the app was twice that reported in Levin et al. (2022).

One reason for high usage and a lack of drop-off of use might be that our study was relatively short; two weeks might not be enough for the novelty aspect of the app to wear out. Another could be that participants indeed perceived the mental health value of the app, especially for anxiety and stress, which are, as mentioned above, big issues on college campuses right now. The daily measurements may have made the progress visible to our participants and thus worked as an additional motivator. A third potential reason could be more indirectly related to the daily probes. Previous work has shown that probing participants to use a meditation app resulted in more frequent access (Militello et al., 2022). Our probes were data-collection probes and did not explicitly remind participants to use the app, but they might have indirectly served as such reminders. A fourth potential reason for the lack of drop-off could be that we explicitly recruited participants for a study about a mindfulness app, which likely created an obvious self-selection bias towards participants who are intrinsically motivated to use such an app. This, however, is likely true for other such interventions as well. Finally, perceived coercion is unlikely to be at play, because participants were completely free to choose how often and when or even whether to access the app; course credit was not dependent on frequency of access.

Participants generally had a favorable view of the app, rating it highly for function and aesthetics, above average for engagement, and around average for subjective quality, as expected from the literature (see Mani et al., 2015, for a review). Usage correlated positively with the engagement and function ratings, implying either or both that people who used the app found it fun and functional, or that people who found the app fun and functional used it more often. Subjective quality (i.e., whether one would recommend the app to others, use it frequently, or be willing to pay for it) did not correlate with usage. Note that if we assume that the functionality and engagement value of the app drive usage, one might be tempted to conclude that a simple way to encourage more frequent or longer usage would be to optimize these aspects of the app. Unfortunately, participants’ ratings of the app were already quite high, so there seems to not be a lot of room for improvement on that front.

Some practical considerations can be derived from this study. First, changes in state mindfulness are mediators between app use and changes in state anxiety, depression and stress. This suggests that any intervention that aims at positive changes in mental health might benefit from including or emphasizing mindfulness-related content.

Another consequence is that mindfulness itself might be a good target for intervention. Apps such as Headspace use meditation to temporarily elevate levels of mindfulness. Not everyone, however, enjoys meditation, and therefore alternative approaches, such as emphasizing mindfulness practices in everyday life might be worth exploring. In that vein, in a one-year follow-up to a successful mindfulness intervention in college students, Galante et al. (2021) found that effects on stress were maintained in those who continued using informal mindfulness exercises, apart for meditation. A recent intervention that utilizes no meditation is the Mindfulness Ecological Momentary Intervention (Zainal & Newman, 2023). Interventions promoting mindfulness in daily life are still scarce, and this constitutes a gap in the health and well-being app world, as well as in mindfulness research in general.

Second, we note that app use was fairly high in our group of participants, with only implicit encouragement needed. This begs the question as to why these participants were not using the app already—it was, after all, freely available on campus and advertised as such by our well-being programs. Possibly more active promotion of the app on campus, perhaps with explicit references to the research showing its effectiveness, might be useful.

### Limitations and further research

Our study suffers from a number of limitations. One is the short duration of the assessment phase. A longer app use phase would be useful for looking at reasons for drop-out or drop-off in the longer term. It would also allow us to investigate whether the lack of translation from state to trait in the present data set is due to insufficient experience with meditation. A second limitation is that the time course of effects cannot unambiguously be ascribed to app use without a control group. A third limitation is that participants used simple unidimensional sliders to assess state mindfulness, depression, anxiety and stress. We see this as both a strength and a limitation—the former because such idiosyncratic assessment is likely more ecologically valid, and plausibly taps into aspects of these outcomes participants find meaningful; the latter because it is unclear how these potentially highly personal definitions map onto more clinical definitions of these concepts. Fourth, our participants self-selected into this study, which limits the generalizability of effects and mechanisms to those who are intrigued by such apps in the first place. To wit, a large number (43%) of participants indicated having used meditation app before, demonstrating some prior interest, motivation and willingness to engage with the app and with mindfulness more generally, at least in this subsample. Fifth, the population consisted entirely of college students enrolled in psychology classes (and thus presumably with an interest in how the mind functions); it is not clear whether the results would generalize to the population at large. Sixth, we investigated only a single app, and it is not clear if the results would generalize to other mindfulness or meditation apps. Finally, we did not have access to the participants’ Headspace records, potentially creating noise in the data and/or reporting bias. We note that reporting bias would go against hypothesis (i.e., if participants did not meditate, but indicated they did, and if meditating correlated with state mindfulness, this would decrease the observed correlation; the same is true for the opposite bias). Finally, our response time window for the daily surveys was open for 6 h; this may have introduced additional noise in the data.

These limitations offer obvious windows into possible venues for further research. Additionally, investigating ways to get college students and other individuals to actually take the first step towards using these apps as well as investigating ways to increase app usage would be useful. We are also intrigued by the potential conclusion that app use might lead to cumulative effects on mental health, even when state mindfulness per se does not change over the course of the intervention. A more formal investigation into this finding would be welcome. Finally, it would be interesting to investigate if and how in-person support for or assistance with the app could potentially enhance the effects on mental health and well-being.

## Data Availability

The data are accessible via Open Science Framework at https://osf.io/vjcf4/
